# Entanglement Characteristic Time from Complex Moduli via i-Rheo *GT*

**DOI:** 10.3390/polym14235208

**Published:** 2022-11-30

**Authors:** Dongdong Li, Lukun Feng, Yin Tang, Caizhen Zhu

**Affiliations:** Institute of Low-Dimensional Materials Genome Initiative, College of Chemistry and Environmental Engineering, Shenzhen University, Shenzhen 518060, China

**Keywords:** molecular dynamic simulation, entanglement dynamics, stress relaxation

## Abstract

Tassieri et al. have introduced a novel rheological tool called “i-Rheo *GT*” that allows the evaluation of the frequency-dependent materials’ linear viscoelastic properties from a direct Fourier transform of the time-dependent relaxation modulus *G*(*t*), without artifacts. They adopted i-Rheo *GT* to exploit the information embedded in *G*(*t*) derived from molecular dynamics simulations of atomistic and quasi-atomistic models, and they estimated the polymers’ entanglement characteristic time ($\tau_{e}$) from the crossover point of the moduli at intermediate times, which had never been possible before because of the poor fitting performance, at short time scales, of the commonly used generalized Maxwell models. Here, we highlight that the values of $\tau_{e}$ reported by Tassieri et al. are significantly different (i.e., an order of magnitude smaller) from those reported in the literature, obtained from either experiments or molecular dynamics simulations of different observables. In this work, we demonstrate that consistent values of $\tau_{e}$ can be achieved if the initial values of *G*(*t*), i.e., those governed by the bond-oscillation dynamics, are discarded. These findings have been corroborated by adopting i-Rheo *GT* to Fourier transform the outcomes of three different molecular dynamics simulations based on the following three models: a dissipative particle dynamics model, a Kremer–Grest model, and an atomistic polyethylene model. Moreover, we have investigated the variations of $\tau_{e}$ as function of (i) the ‘cadence’ at which *G*(*t*) is evaluated, (ii) the spring constant of the atomic bone, and (iii) the initial value of the shear relaxation modulus *G*(O). The ensemble of these results confirms the effectiveness of i-Rheo *GT* and provide new insights into the interpretation of molecular dynamics simulations for a better understanding of polymer dynamics.

## 1. Introduction

The stress relaxation function, $G\left(t\right)$, of the entangled polymer melts in the linear regime is one of the most fundamental and important dynamic properties in the tube theory. Since the efficient multiple-tau correlator method was proposed [1,2], the linear stress relaxation data with good quality become accessible from the molecular dynamics (MD) simulations. In order to reveal the linear viscoelastic behaviors, $G\left(t\right)$ is generally transformed into the storage and loss moduli $G^{\prime }\left(\omega \right)$ and $G^{\prime \prime }\left(\omega \right)$ by fitting $G\left(t\right)$ with the multi-mode Maxwell function. However, this procedure may suffer from some uncertainties and artifacts. Recently, Tassieri et al. proposed an effective analytical tool called “i-Rheo *GT*” to convert the time-dependent $G\left(t\right)$ into the frequency-dependent complex moduli, $G^{\prime }\left(\omega \right)$ and $G^{\prime \prime }\left(\omega \right)$, via the oversampling technique, wherein the raw $G\left(t\right)$ data are interpolated to produce oversampled data [3]. Since i-Rheo *GT* does not require any preconceived models, the artifacts, especially in the high-frequency regime which may exist in the early works, could be ruled out. For example, for the generic Kremer–Grest (KG) model, a crossover of $G^{\prime }\left(\omega \right)$ and $G^{\prime \prime }\left(\omega \right)$ at the high frequency like in the vicinity of $1/\tau_{e}$ with $\tau_{e}=N_{e}{}^{2}\zeta b^{2}/\left(3\pi^{2}k_{B}T\right)$ where $\tau_{e}$ is the entanglement time, $N_{e}$ is the entanglement length, ζ is the monomeric friction coefficient, b is the Kuhn segment length, and T is the temperature, is observed for the first time [3]. Thus, a specific problem/challenge, that is, the qualitative disagreement between the fitted $G^{\prime }\left(\omega \right)$ and $G^{\prime \prime }\left(\omega \right)$ from the multi-mode Maxwell function fittings of $G\left(t\right)$ with the experimental data is overcome, indicating that a direct determination of $\tau_{e}$ from the crossover becomes possible with i-Rheo *GT* for the KG model. Furthermore, when applying i-Rheo *GT* to the atomistic polyethylene (PE) model, a dependence of $\tau_{e}$ on the molecular weight, M, (or the chain length, N) is found, for the first time, to be in a stretched exponential form which approaches a constant value from above. Note that the convergence performance of $\tau_{e}\left(M\right)$ (or $\tau_{e}\left(N\right)$), which approaches an asymptotic constant value from bigger values with an increase in M, is qualitatively similar with the dependence of $N_{e}$ on the chain length N wherein $N_{e}$ is obtained from the modified estimators defined in the primitive path analyses (PPA) [4]. Since its emergence, i-Rheo *GT* has been applied in different systems (e.g., ring catenane, polymer-elastomer blends, elastomeric networks, and hierarchical simulation) [5,6,7,8] for the evaluation of the viscoelastic properties and has been implemented in the REPTATE rheology software [9].

However, the $\tau_{e}$-values of both coarse-grained (CG) KG and atomistic PE models estimated from i-Rheo *GT* seem inconsistent with previously reported results [3]. For the CG KG model, $N_{e}$ calculated from PPA, plateau modulus in the stress relaxation, and the monomer mean-square-displacement (MSD) is about 85 [10,11,12]. Correspondingly, $\tau_{e}\approx 10^{4}\;\tau_{\mathrm{LJ}}$ where $\tau_{\mathrm{LJ}}$ is the Lennard-Jones (LJ) time unit. In addition, when fitting $G\left(t\right)$ with the slip-links model, $\tau_{e}\approx 5800\;\tau_{\mathrm{LJ}}$. While $\tau_{e}$ estimated from i-Rheo *GT* is about $671\tau_{\mathrm{LJ}}$, which is approximately one order of magnitude smaller than those estimated by other methods mentioned above. On the side, for the atomistic PE model, the value of $\tau_{e}\approx 0.32\;\mathrm{ns}$ (at 450 K) obtained from the complex moduli via i-Rheo *GT* is somewhat smaller than those obtained from MSD, rheology theory mapping, and experiments [3,13]. Especially with the same definition of $\tau_{e}$ as Tassieri et al. [3], Szántó et al. [14] utilized the high-frequency rheometer to experimentally determine $\tau_{e}$ and obtained a $\tau_{e}$ of $3.0_{-1.8}^{+9.0}$ ns (at 463 K). The lower bound of the experimental $\tau_{e}$ is still almost four times larger than that from Tassieri et al. via i-Rheo *GT* [3]. Apparently, the large discrepancies of $\tau_{e}$ estimated from i-Rheo *GT* and other methods, including simulations and experiments, could not be simply attributed to different physical observables. We note that $N_{e}$ of the KG model, which is estimated from PPA, stress relaxation, and MSD, has reached consistent results [12,13], despite the fact that PE may exhibit some complex effects of the chemical structure on entanglement characters of the melts [1,15]. Since $\tau_{e}$ is one of the most fundamental parameters in the tube theory and all viscoelastic polymers, it is meaningful to clarify the discrepancy of $\tau_{e}$ calculated from i-Rheo *GT* and other methods and find an approach to ensure consistency of $\tau_{e}$ estimated from different methods. In this work, for a comprehensive study of the above important and fundamental issues in polymer science, we conducted the hierarchical molecular dynamics simulations at different scales and investigated the linear rheology in the entangled systems. Three typical model systems are considered, including models with high computational efficiency to obtain strongly entangled polymer melts at a reasonable computational cost and models for which extensive detailed studies and relevant reference data are available. The simulated models include the entangled dissipative particle dynamics (DPD) model, generic CG KG model [16], and atomistic PE model [17]. For the computationally efficient DPD model [18,19,20,21], the polymer melts with different chain lengths are simulated. For KG and PE models, only well-entangled melts are simulated.

## 2. Simulation Details

### 2.1. DPD Simulation

In DPD, all beads interact with a pairwise conservative force ($F^{C}$), dissipative force ($F^{D}$), and random force ($F^{R}$). For example, the forces between bead i and j located at the coordinates of $r_{i}$ and $r_{j},$ respectively, are given below:

$$F_{ij}^{C}=a_{ij}\left(1-\frac{r_{ij}}{r_{c}}\right){\hat{r}}_{ij}\;\mathrm{for}\;r_{ij}\leq r_{c},\;\mathrm{or}\;0\;\mathrm{for}\;r_{ij}>r_{c}$$

$$F_{ij}^{D}=-\gamma {\left(1-\frac{r_{ij}}{r_{c}}\right)}^{2}\left(v_{ij}\cdot {\hat{r}}_{ij}\right){\hat{r}}_{ij}$$

$$F_{ij}^{R}=\frac{\sigma \zeta_{ij}}{\sqrt{\Delta t}}\left(1-\frac{r_{ij}}{r_{c}}\right){\hat{r}}_{ij}$$

where $r_{ij}=r_{i}-r_{j}$, $r_{ij}=\left|r_{ij}\right|$, ${\hat{r}}_{ij}=r_{ij}/r_{ij}$, $v_{ij}=v_{i}-v_{j}$, $a_{ij}$ is the repulsion constant, $r_{c}$ is the cutoff radius, γ is the friction parameter, $v_{i}$ is the velocity of bead i, σ is the thermal noise amplitude, $\zeta_{ij}$ is the Gaussian random variable, and Δt is the simulation timestep. Note γ and σ are connected according to the fluctuation-dissipation relaxation: $\sigma^{2}=2\gamma k_{B}T$ with $k_{B}$ being the Boltzmann constant and T being the temperature. The sequential beads in the same chain are connected by the harmonic springs:

$$F_{ij}^{B}=K_{B}\left(l-r_{ij}\right){\hat{r}}_{ij}$$

with $K_{B}$ being spring constant and l being equilibrium bond length. Additionally, a bond bending potential taken as $U_{bend}\left(\theta \right)=K_{\theta }\left(1+cos\theta \right)$ where $K_{\theta }$ is the bending stiffness constant, and θ is the angle of consecutive bond vectors, is added to increase the stiffness of chain. The quantities are taken in the DPD reduced units, namely, the units of length, mass, and energy are set as $r_{c}$, m, and $k_{B}T$ respectively. The corresponding time unit is $\tau =r_{c}{\left(\frac{m}{k_{B}T}\right)}^{1/2}$. It is worth stressing that, different from the standard DPD, the conservative force used here is properly increased to prevent chain crossings from showing entanglement effects [22]. The parameters of the DPD model used here are the same with refs [18,19,20,21] in which $a_{ij}=200$, $r_{c}=1$, γ=4.5, Δt=0.012, $k_{B}T=1$, $K_{B}=400$, l=0.95, $K_{\theta }=2$, and the density ρ=1 in DPD reduced units.

### 2.2. KG Simulation

In the standard KG model [16], the bead-spring polymer chains are represented as a sequence of beads connected by the finitely extensible nonlinear elastic (FENE) spring. All beads interact by the purely repulsive Lennard-Jones (LJ) potential:

$$U_{LJ}\left(r\right)=\left\{\begin{array}{c} 4\varepsilon \left[{\left(\frac{\sigma }{r}\right)}^{12}-{\left(\frac{\sigma }{r}\right)}^{6}+\frac{1}{4}\right],r<r_{c}\\ 0,r\geq r_{c} \end{array}\right.$$

where r is the distance between two beads, ε is the LJ energy scale, and σ is the LJ radius. The cutoff distance $r_{c}$ is set as $2^{1/6}\sigma$. The FENE potential is described as:

$$U_{FENE}\left(r\right)=-\frac{1}{2}kR_{0}{}^{2}\ln\left(1-\frac{r^{2}}{R_{0}{}^{2}}\right)$$

where k is the spring constant and equals 30$\varepsilon /\sigma^{2}$, $R_{0}$ = 1.5σ is the maximum bond length. All quantities are expressed in the LJ reduced units. σ, ε, and the bead mass m are equal to 1. The temperature is maintained at $T=\varepsilon /k_{B}$ where $k_{B}$ is Boltzmann’s constant. The corresponding time unit is $\tau_{\mathrm{LJ}}={\left(m\sigma^{2}/k_{B}T\right)}^{0.5}$. For our MD simulations of the KG model, the simulation parameters are the same as those of Tassieri et al. [3], except for the thermostat and timestep. We note that compared to the Langevin thermostat used in the work of Tassieri et al. [3], the DPD thermostat could conserve both global and local momentums. Therefore, in the present work, the DPD thermostat is used (instead of the Langevin thermostat), and the timestep is $\Delta t=0.006\tau_{\mathrm{LJ}}$ (other than $0.012\tau_{\mathrm{LJ}}$).

### 2.3. PE Simulation

In the case of PE, the well-known TraPPE united-atom (UA) force field is used [17]. Each terminal methyl CH_3_ group and internal methylene CH_2_ group is set as a single interacting point. The bonded interactions include bond stretching, bond angle bending, and torsional rotation. In the original description of the TraPPE force field, the bond lengths are fixed at 1.54Å. In this work, the harmonic bond potential of $U=K{\left(r-r_{0}\right)}^{2}$ with the spring constant of K=120 kcal/mol and the equilibrium bond length of $r_{0}=1.54Å$ is taken. The bond angle bending potential is described by a harmonic potential:

$$U_{bend}\left(\theta \right)=\frac{1}{2}k_{\theta }{\left(\theta -\theta_{0}\right)}^{2}$$

where $k_{\theta }$ is the force constant with $k_{\theta }/k_{B}$ = 62,500 K rad^−2^ and the equilibrium angle $\theta_{0}$ is $114^{{}^{\circ}}$. The torsional interaction is governed by the OPLS (optimized potentials for liquid simulations) potential:

$$U_{torsion}\left(\phi \right)=c_{1}\left[1+\cos\phi \right]+c_{2}\left[1-\cos\left(2\phi \right)\right]+c_{3}\left[1+\cos\left(3\phi \right)\right]$$

where the force constants $c_{1}$, $c_{2}$ and $c_{3}$ are 355.03 $k_{B}$K, −68.19 $k_{B}$K, and 791.32 $k_{B}$K, respectively. The non-bonded interactions are described by LJ potentials:

$$U_{LJ}\left(r\right)=4\varepsilon \left[{\left(\frac{\sigma }{r}\right)}^{12}-{\left(\frac{\sigma }{r}\right)}^{6}\right]$$

where the parameters of CH_3_ and CH_2_ groups are $\varepsilon_{{\mathrm{CH}}_{3}}/k_{B}=104$K, $\varepsilon_{{\mathrm{CH}}_{2}}/k_{B}=46$K, $\sigma_{{\mathrm{CH}}_{3}}=3.91Å$ and $\sigma_{{\mathrm{CH}}_{2}}=3.95Å$, respectively. The interactions between unlike particles are computed by Lorentz-Berthelot mixing rules. The cutoff distance of LJ interactions is 14Å.

## 3. Results and Discussion

### 3.1. DPD

**Figure 1:**
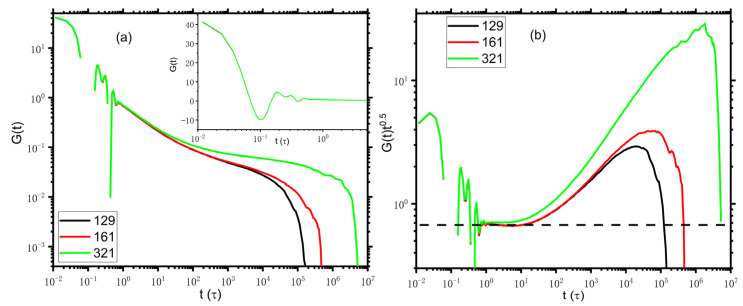
(**a**) Stress relaxation for DPD polymer chains with N = 129, 161, and 321. The inset shows the bond length relaxation regime at an early time for N = 321. (**b**) The same with panel (**a**), but the vertical coordinate is normalized by the power law of the Rouse dynamics regime $t^{0.5}$. The horizontal dash line indicates the pure Rouse behavior.

We first focus on the DPD model as an alternative to the entanglement molecular model for MD simulations, which, as noted above, has become a promising tool for studying the long-time dynamics of polymeric liquids. In Figure 1, we present the stress relaxation for the DPD polymer chains with lengths N=129, 161, and 321. The inset shows the bond oscillations at the early time in a semi-logarithmic plot. The stress is calculated at every timestep via the multiple-tau correlator algorithm [1,2]. The stress relaxation behaviors of the DPD model are qualitatively similar to those of the KG model. Note that as indicated by the horizontal dash line in Figure 1b, the normalized stress relaxation, $G\left(t\right)t^{0.5}$, shows a plateau as expected from the Rouse model for t after the bond length relaxation. However, the slope of $G\left(t\right)t^{0.5}$ for the standard KG model at 1<t<30 is about −0.13, which some deviates from the prediction of the Rouse model [1]. In this regard, the DPD model seems more suitable to describe stress relaxation.

**Figure 2:**
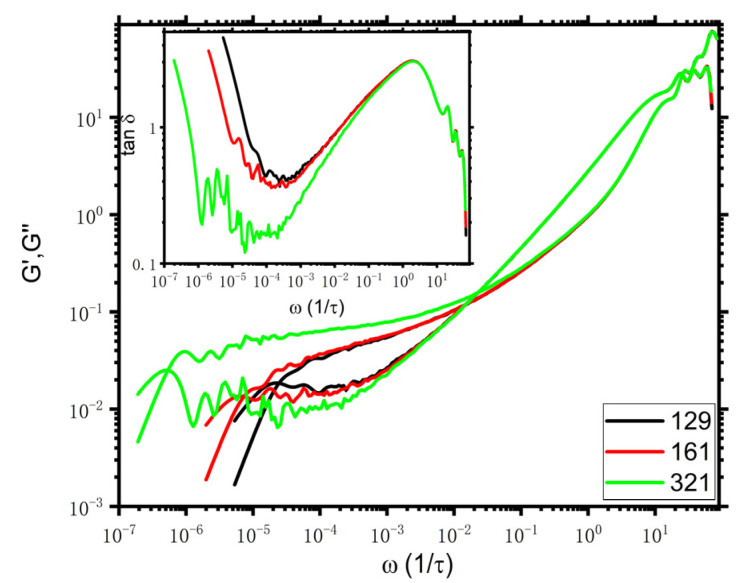
Viscoelastic moduli for N=129, 161, and 321. The absolute values of $G\left(t\right)$ are input into i-Rheo *GT* to obtain the viscoelastic moduli, and the values of $G\left(0\right)$ are 43.7, 43.7, and 43.8 for N=129, 161, and 321, respectively. The tanδ vs. frequency is presented in the inset.

The resulting viscoelastic moduli data via i-Rheo *GT* by feeding the absolute values of $G\left(t\right)$ into i-Rheo *GT,* as suggested in Ref. [3], are shown in Figure 2. Note that the quality of the curves of N=321 are not as good as those of N=129 and 161 since the total simulation times of the latter ones are almost 200 times longer than their disentanglement time while the simulation time of the former one is less than a decade of its disentanglement time. Nevertheless, Figure 2 distinctly indicates a crossover point of the storage elastic modulus $G^{\prime }$ and the loss of viscous modulus $G^{\prime \prime }$ at $\omega_{\mathrm{cross}}$ in the intermediate frequency scale for all systems. Furthermore, as presented in the inset of Figure 2, $\tan\delta \left(10\omega_{\mathrm{cross}}\right)\approx 2$, which is in agreement with the theory and experiments [3]. We could further estimate $\tau_{e,\;GT}$ from $1/\omega_{\mathrm{cross}}$ where the subscript GT in $\tau_{e,GT}$ indicates the entanglement time is obtained from i-Rheo *GT*. $\tau_{e,GT}$ are about 68, 67, and 44 for N=129, 161, and 321, respectively, much smaller (beyond an order of magnitude) than the entanglement time estimated from MSD, which is indicated as $\tau_{e}^{*}\approx 810$ or $\tau_{e}=\frac{9\tau_{e}^{*}}{\pi }\approx 2300$ given in Figure S1 in SI. This finding is similar to the results of KG and PE models reported by Tassieri et al. [3], as mentioned above. Additionally, the smaller value $\tau_{e,\;GT}$ of N=321 than those of the other two shorter counterparts indicates that $\tau_{e,\;GT}$ relies on the chain length, as further evidenced by the broad chain length window in Figure S2 in SI, which qualitatively agrees with the results of PE by Tassieri et al. [3] as well.

**Figure 3:**
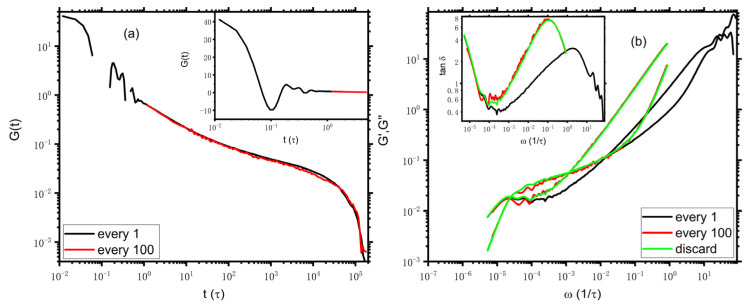
Stress relaxation (**a**) and viscoelastic moduli (**b**) of N=129. The absolute values of $G\left(t\right)$ are input into i-Rheo *GT*, and the value of $G\left(0\right)$ is 43.7. The inset in panel (**b**) presents the loss tangent tanδ vs. frequency. The legends of “every 1” and “every 100” indicate the stress is calculated using every first and hundredth point, respectively. The legend of “discard” means the stress is calculated at every timestep while the data at early time (t<1.2τ) is artificially discarded.

Experimentally, the highest sampling frequency of the rheometers is limited in the mega-hertz range and is usually quite smaller than that taken in molecular simulations, wherein the stress is often sampled every timestep, and the corresponding sampling frequency is in the tera-hertz range [3,14]. Therefore, it is interesting to check the effects of the limited dynamic window as accessed by experiment instruments on the Fourier transformation of $G\left(t\right)$ via i-Rheo *GT*. We now discard the short time data of $G\left(t\right)$, corresponding to the regime of the bond length relaxation, during the transformation from $G\left(t\right)$ to $G^{\prime }\left(\omega \right)$ and $G^{\prime \prime }\left(\omega \right)$ just as early works in experiments and simulations. [1,9] We note that for the DPD model used here, the bond length relaxation regime would be “naturally” discarded if the stress is calculated every hundred timesteps since the corresponding sampling time interval is $1.2\left(=100\Delta t\right)\tau$, with Δt being the simulation timestep and τ being DPD time unit, which is larger than the relaxation time of the bond oscillations, i.e., about 1τ as indicated by the inset of Figure 1. Taking N=129 as an example, as demonstrated in Figure 3a, the resulting $G\left(t\right)$ curves from stress data calculated every timestep and every hundred timesteps (denoted as “every 1” and “every 100”, respectively) fall practically on top of each other, though $G\left(t\right)$ of “every 100” is with poorer quality. As an alternative route to discarding the high-frequency domain of bond length relaxation, we can “artificially” discard the $G\left(t\right)$ data at an early time, i.e., $G\left(t<1.2\right)$, while stress and the original $G\left(t\right)$ are calculated at every timestep, which is denoted as “discard” in what follows. As expected, in Figure 3b, the viscoelastic spectrums from $G\left(t\right)$ of “every 100” nearly overlap with those of “discard”. Since the moduli of “discard” are of better quality than the moduli of “every 100”, for a better understanding of the limited dynamic window problem, we would focus on comparisons of the moduli of “every 1” and “discard” later. As indicated by the black and green lines, though the $G^{\prime }$ curves of “every 1” and “discard” lie on top of each other for ω<0.02, $G^{\prime \prime }$ of the former is smaller than that of the latter apart from ω at the terminal scale, which $G^{\prime \prime }$ is in the same magnitude as both “every 1” and “discard”. Consequently, the cross point of the moduli of “discard” moves to a lower frequency regime. Interestingly, the resulting $\tau_{e}$ is about 820, which is approximately an order of magnitude larger than that estimated from the moduli of “every 1” but very close to $\tau_{e}^{*}\approx 810$ estimated from MSD, as mentioned above. In this regard, the result of discarding the early bond oscillation regime appears to be more consistent with results from other common physical observables, independent of the “naturally” or “artificially” discarding approach used. As we see below, this also holds true for both KG and PE models. A consistent result could be anticipated when i-Rheo *GT* is used to analyze the experimental data since the time scale corresponding to the bond length relaxation is hardly accessible and is usually not included in the rheological experiments. Additionally, as demonstrated by the black and red lines in Figure 3, it seems very tricky that for i-Rheo *GT,* the sampling time interval plays a critical role in the determination of $\tau_{e}$, even though the time interval is still smaller than $\tau_{e}$ by tens of times. We note that the time scale corresponding to the bond length relaxation is beyond the consideration of tube theory on the viscoelastic properties of the polymers [15], while in real polymer melts, there exists an intricate interplay between bonded and non-bonded interactions. The method of i-Rheo *GT* to process the data of the coupling interactions between the different dynamics mechanisms at different time regimes might be the origin of the observed deviation. This, to some extent, is qualitatively similar to the case in which single-chain models obey the stress-optical law rather well for times longer than the bond length relaxation time, whereas for many-chain models, it is valid on slightly longer time scales as a consequence of complicated coupling of the interactions. [23] Nevertheless, we note that as shown in the inset, for the moduli discarding the bond oscillation regime, $\tan\delta \left(10\omega_{\mathrm{cross}}\right)\approx 3.3,$ which disagrees with the theoretical and experimental expectation.

**Figure 4:**
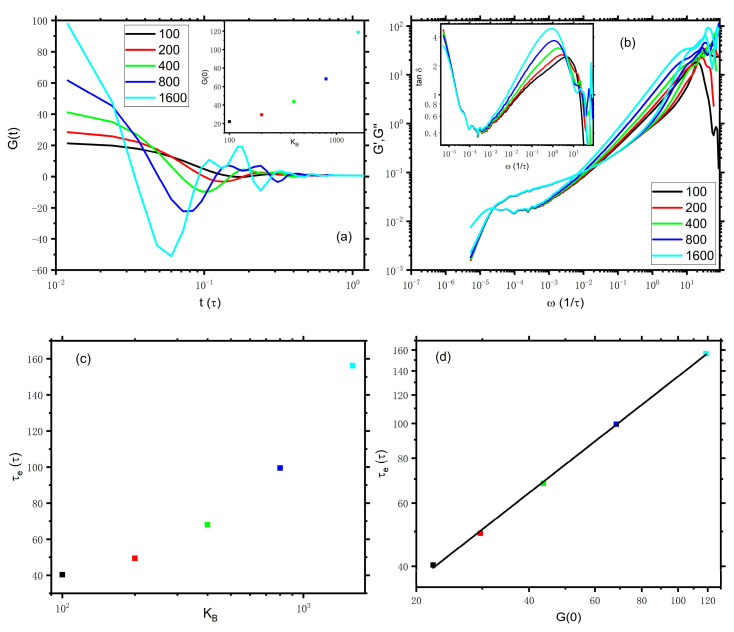
(**a**) Stress relaxation of N=129 for different spring constants, $K_{B}$, ranging from 100 to 1600 at an early time. The value of $G\left(0\right)$ is shown in the inset. (**b**) The corresponding viscoelastic moduli. The absolute values of $G\left(t\right)$ are input into i-Rheo *GT*. The inset presents the tanδ vs. frequency curves. (**c**) Dependence of the entanglement time $\tau_{e}$ on the spring constant $K_{B}$. (**d**) Dependence of the entanglement time $\tau_{e}$ on the values of $G\left(0\right)$ of different spring constant.

The results discussed above show whether the inclusion of the bond length relaxation regime or not plays an important role in the exact determination of relevant parameters in rheology via i-Rheo *GT*. To gain a detailed insight into the influences of the bond length relaxation regime on the determination of $\tau_{e},$ we do further analysis on the “discard” data. For simplicity, we tune the spring constant, $K_{B}$, of bonded beads and artificially replace the data of G(t<1.2) by the ones from DPD simulations with different spring constants while the data of $G\left(t\geq 1.2\right)$ remain unchanged. Note that a smaller $K_{B}$ would result in the crossability of chains and a larger $K_{B}$ requires a smaller timestep [22]. Here, the stress is calculated at every time of 0.012τ though the timestep is different for different $K_{B}$. Taking N=129 as an example again, the early oscillations of bond relaxation for different spring constants, which range from 100 to 1600, are presented in Figure 4a. The height and number of peaks increase with the increase of the spring constant, indicating that the oscillations become more intense and complicated. Particularly, the value of $G\left(0\right)$ increases quickly with the spring constant, as demonstrated in the inset of Figure 4a. Figure 4b presents the corresponding viscoelastic moduli. Although the $G^{\prime }(\omega <0.3)$ curves of different spring constants lie on top of each other, $G^{\prime \prime }$ generally increase with the increase in the spring constant for $\omega >10^{-3}$. The different responses of $G^{\prime }$ and $G^{\prime \prime }$ to the spring constant are similar to the responses of $G^{\prime }$ and $G^{\prime \prime }$ to the sampling frequency as mentioned above. Hence, the values of ω for the cross point become smaller with the increase in the spring constant. The resulting $\tau_{e}$ depends strongly on the spring constant $K_{B}$ as presented in Figure 4c, even though $G\left(t\geq 1.2\right)$ are the same for all systems with different spring constants. Additionally, we would like to point out that $\tau_{e}$ even shows an exponential dependence on $G\left(0\right)$, which can be described by a power law of $\tau_{e}\propto G{\left(0\right)}^{0.8}$ as demonstrated in Figure 4d. These findings convincingly demonstrate that the dynamics at the shorter time scale could intensively affect the calculation of $\tau_{e}$ located at a longer (at least by dozens of times) scale when applying i-Rheo *GT* to convert $G\left(t\right)$ into $G^{\prime }\left(\omega \right)$ and $G^{\prime \prime }\left(\omega \right)$, while the same $\tau_{e}$ is expected to be obtained if we “artificially” discard the data of G(t<1.2). Altogether, our DPD results reveal the limits of applicability of i-Rheo *GT* to the exact determination of relevant parameters in rheology. The reason for this apparent failure lies in the method of i-Rheo *GT* to process the data of the coupling interactions between the different dynamic mechanisms at different time regimes.

### 3.2. KG

**Figure 5:**
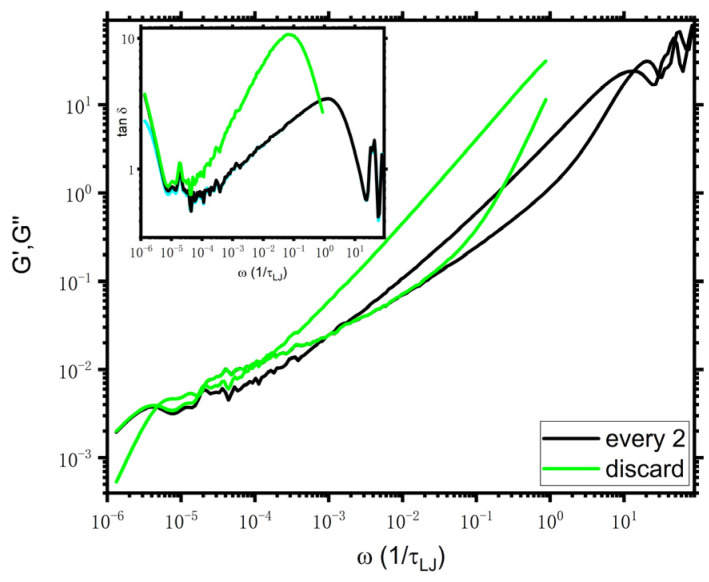
Viscoelastic moduli of KG model. The absolute values of $G\left(t\right)$ are input into i-Rheo *GT*, and the value of $G\left(0\right)$ is 67.7. The inset presents the tanδ vs. frequency. The legends of “every 2” and “discard” mean that the stress is calculated using every second point, and the data of G(t<1.2) of “every 2” are discarded, respectively.

To elucidate the above issues further, we consider KG model as another test case and check the consistency of $\tau_{e}$ estimated from i-Rheo *GT* with those available reference data from other methods. The chain length is N=350, which is the same as the longest chain of Tassieri et al. [3] Note that the stress is calculated every two timesteps here to keep the same sampling time interval with the work of Tassieri et al. [3] since the sampling frequency seems to play a role in i-Rheo *GT* analysis as mentioned above. The resulting viscoelastic moduli of the KG model are shown in Figure 5. The curves of “every 2” are generally the same as those in the work of Tassieri et al. [3] despite that the quality of the curves at the middle scale ($\omega \approx 10^{-5}-10^{-4}/\tau_{LJ}$) in this work is poorer. An approximate range of $\tau_{e,GT}$ estimated from $\tan\delta \left(\omega_{cross}\right)=1$ (as shown in the inset of Figure 5) is 600~900, which is comparable with the result of 671 reported by Tassieri et al. [3] but is quite smaller than those calculated by other methods as mentioned above. Additionally, the characteristic time for tanδ=2 is about 25, which is smaller than $\tau_{e,GT}$ by several tens of times. Consequently, the expectation of $\tan\delta \left(10\omega_{cross}\right)=2$ is not satisfied from the results of “every 2” for the KG model (this expectation is met for the DPD model). Like in the DPD case, we “artificially” discard the $G\left(t\right)$ data at an early time, i.e., G(t<1.2). As indicated by the green curves in Figure 5, the behaviors of $G^{\prime }$ and $G^{\prime \prime }$ are similar to those of the DPD model described above, namely, the curve of $G^{\prime }(\omega <0.02)$ of “discard” falls on top of that of “every 2” and $G^{\prime \prime }$ is on the top of that of “every 2”, except for the terminal scale. $\tau_{e,GT}$ estimated from tanδ=1 of “discard” is $\tau_{e,GT}\approx 8400$, which is comparable with the values from other estimators as mentioned above, i.e., $\tau_{e}^{*}$ estimated from MSD for the KG model is $\tau_{e}^{*}\approx 2950$ [24], thus $\tau_{e}=9\tau_{e}^{*}/\pi \approx 8450$. Nevertheless, the expectation of $\tan\delta \left(10\omega_{cross}\right)=2$ is not satisfied from the results of “discard” as well.

### 3.3. PE

**Figure 6:**
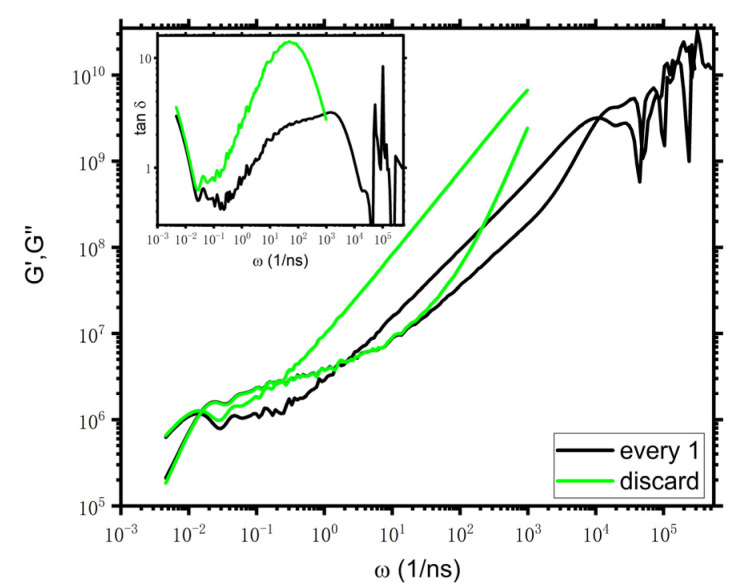
Viscoelastic moduli of PE. The absolute values of $G\left(t\right)$ are input into i-Rheo *GT*, and the value of $G\left(0\right)$ is 14.5 GPa. The inset shows tanδ vs. frequency. The legends of “every 1” and “discard” mean that the stress is calculated at every timestep, and the data of $G(t<10^{-3}\mathrm{ns})$ of “every 1” are discarded, respectively.

For a complete understanding of the limits of applicability of i-Rheo *GT*, we then consider the well-studied atomistic PE model. The MD simulation condition is the same as the work of Tassieri et al. [3] (at 450 K, 1 atm). The stress is calculated at every timestep $\left(\Delta t=2\;\mathrm{fs}\right)$. The molecular weight is 5.04 kg/mol, corresponding to N=360 carbon atoms per chain. Figure 6 presents the viscoelastic moduli of PE. Similar to the results of DPD and KG models, the curves of $G^{\prime }$ of “every 1” and “discard” lie on top of each other as ω<20/ns while the curves of $G^{\prime \prime }$ of these two cases overlap with each other only at the terminal regime. $\tau_{e,GT}$ are approximately estimated to be 0.55 ns and 4.2 ns from “every 1” and “discard”, respectively. Note that these two $\tau_{e,GT}$ are separately compared with the value calculated from i-Rheo *GT* (which is 0.37 ns for the molecular weight being 5.04 kg/mol) and the value of ~5.5 ns estimated from MSD in Ref. [3]. Consistent with DPD and KG models, $\tau_{e,GT}$ estimated from “discard” here is in better agreement with $\tau_{e}$ from other methods, however, $\tau_{e,GT}$ from “every 1” appears to be somewhat smaller. As for $\tan\delta \left(10\omega_{cross}\right)$, the values are about 2.1 for “every 1”and 4.4 for “discard”, which is similar to the case of DPD.

**Figure 7:**
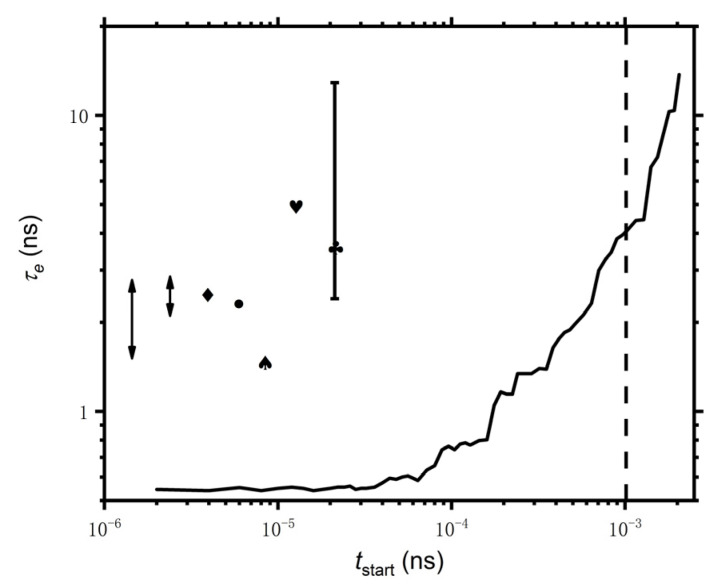
Dependence of $\tau_{e}$ of PE model on the start time, $t_{start}$, which means the data of $G(t<t_{start})$ are discarded. $\tau_{e}$ from Table 2 in Ref. [13] are shown on left side. The vertical dash line indicates the end of the bond length relaxation.

To further elaborate on the influence of the high-frequency dynamics, we gradually discard the data from the highest range and the obtained $\tau_{e}$ is presented in Figure 7. For $t_{start}<10^{-4}$ ns, the obtained $\tau_{e}$ remains unchanged and is quite smaller than the reported values shown by the lines and symbols on the left-hand side. The obtained $\tau_{e}$ increases quickly with the increase of $t_{start}$ before the end of bond length relaxation ($10^{-4}<t_{start}<10^{-3}$) and increases even faster after the bond length relaxation. Note that the experimentally accessible time range is quite larger than the relaxation time of bond length fluctuation (~$10^{-3}$ ns). Generally, the resulting $\tau_{e}$ from i-Rheo *GT* would be comparable with the reported ones, which are obtained from MSD, rheology theory mapping, neutron spin-echo spectroscopy (NSE), and rheology experiments if the high-frequency dynamics is discarded, indicating that the ability to interpret the data at the high-frequency dynamics for i-Rheo *GT* seems insufficient in the current framework of the tube theory. It would be very interesting to look forward to improving rheology theory and experiment techniques to interpret high-frequency dynamics.

## 4. Conclusions

In conclusion, although i-Rheo *GT* could transform the stress relaxation from time to frequency domain without preconceived models and over an extremely wide range of frequencies, especially at high scales, the determination of the entanglement time from the transformed complex moduli could not offer a consistent result with those estimated from other common methods but depends on the sampling frequency at least for DPD, KG models and atomistic PE model in the current framework of tube theory. Interestingly, by discarding the stress relaxation data at high frequency upon applying i-Rheo *GT,* the consistency of the resulting entanglement time with those estimated from experiments and simulations is reached. Thus, it is conceivable that the practical approaches for determining entanglement time from $G\left(t\right)$ might be the use of i-Rheo *GT* with discarding the early bond oscillations regime or the fit of $G\left(t\right)$ with well-built models such as tube or slip-links models [1,25].

## Data Availability

Not applicable.

## Supplementary Materials

The following supporting information can be downloaded at: https://www.mdpi.com/article/10.3390/polym14235208/s1, Figure S1: Mean-square displacement of the middle monomers for N=129, 161, and 321. The vertical coordinate is normalized by the power law of $t^{0.25}$. The intersecting time of the time regimes $t<\tau_{e}$ and $t>\tau_{e}$ is indicated as $\tau_{e}^{*}$; Figure S2: tanδ vs. frequency ω for the chain length ranging from 64 to 321. The regime of tanδ around 1 is enlarged in the inset [26,27,28].
