# Supplementary material for: Entanglement Characteristic Time from Complex Moduli via i-Rheo GT
Source: Polymers (Basel). 2022 Nov 30;14(23):5208. doi: 10.3390/polym14235208 (PMC9740520; doi:10.3390/polym14235208)
Supplement: Supplementary file 1 [file polymers-14-05208-s001.zip › polymers-2004072-supplementary.pdf]

Supporting Information

Entanglement Characteristic Time from Complex Moduli via i-Rheo *GT*

Entanglement time τ_e of DPD model from MSD

Figure S1 presents MSD, $g_1(t)$, of the middle monomers in a double-logarithmic scale in which $g_1(t)$ is normalized by $t^{0.25}$. We see that $\frac{g_1(t)}{t^{0.25}}$ for the longest chain shows a plateau-like region, corresponding to the one-dimensional Rouse motion. By fitting the data with straight lines in the different regimes to the scaling laws, namely, $t < \tau_e$ and $t > \tau_e$, the intersecting time is estimated to be about $\tau_e^* \approx 810$ as indicted in Figure S1. The prefactor $\beta = \sqrt{2/\pi}$ is introduced to further calculate the model parameters.[6] The resulting $\tau_e = 9\tau_e^*/\pi \approx 2300$ and $N_e \approx 28$. Note that N_e calculated from MSD is the same with that calculated from the S-coil estimators defined in Z1-code[7-10]. Additionally, for DPD in this work, N_e is about 26.8 ± 2.5 from the classical S-coil estimator and from the modified S-coil estimator.

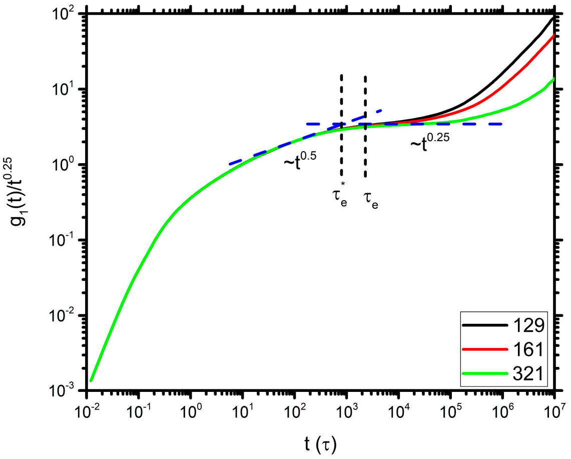

Figure S1. Mean-square displacement of the middle monomers for $N = 129$, 161, and 321. The vertical coordinate is normalized by the power law of $t^{0.25}$. The intersecting time of the time regimes $t < \tau_e$ and $t > \tau_e$ is indicated as τ_e^*.

tan δ vs frequency of different chain length for DPD model

tan δ for three extra chain length, namely, $N = 64$, 80, and 96, is given in Figure S2. At the frequency ω around 0.02 as presented in the inset of Figure S2, tan δ reaches 1 at a slower ω for shorter chains definitely indicating a dependence of the entanglement time on the chain length.

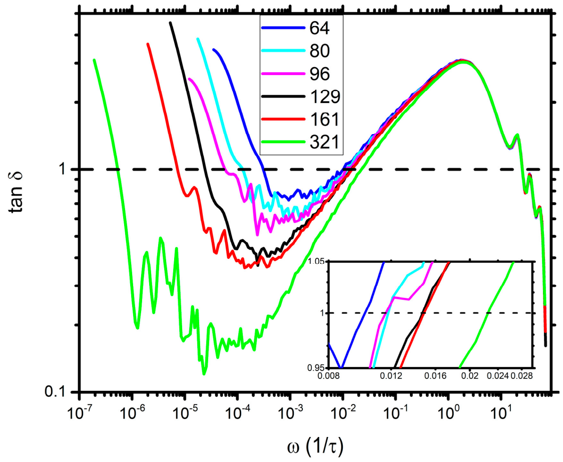

Figure S2. tan δ vs frequency ω for the chain length ranging from 64 to 321. The regime of tan δ around 1 is enlarged in the inset.

References

1. Nikunen, P.; Vattulainen, I.; Karttunen, M., Reptational dynamics in dissipative particle dynamics simulations of polymer melts. *Phys. Rev. E* **2007,** *75* (3), 036713.
2. Mohagheghi, M.; Khomami, B., Molecular Processes Leading to Shear Banding in Well Entangled Polymeric Melts. *ACS Macro Lett.* **2015,** *4* (7), 684-688.

3. Mohagheghi, M.; Khomami, B., Molecularly based criteria for shear banding in transient flow of entangled polymeric fluids. *Phys. Rev. E* **2016,** *93* (6), 062606.
4. Mohagheghi, M.; Khomami, B., Elucidating the flow-microstructure coupling in the entangled polymer melts. Part I: Single chain dynamics in shear flow. *J. Rheol.* **2016,** *60* (5), 849-859.
5. Mohagheghi, M.; Khomami, B., Elucidating the flow-microstructure coupling in entangled polymer melts. Part II: Molecular mechanism of shear banding. *J. Rheol.* **2016,** *60* (5), 861-872.
6. Hou, J.-X., Note: Determine entanglement length through monomer mean-square displacement. *J. Chem. Phys.* **2017,** *146* (2), 026101.
7. Kröger, M., Shortest multiple disconnected path for the analysis of entanglements in two- and three-dimensional polymeric systems. *Comput. Phys. Commun.* **2005,** *168* (3), 209-232.
8. Shanbhag, S.; Kröger, M., Primitive Path Networks Generated by Annealing and Geometrical Methods: Insights into Differences. *Macromolecules* **2007,** *40* (8), 2897-2903.
9. Hoy, R. S.; Foteinopoulou, K.; Kröger, M., Topological analysis of polymeric melts: Chain-length effects and fast-converging estimators for entanglement length. *Phys. Rev. E* **2009,** *80* (3), 031803.
10. Karayiannis, N. C.; Kröger, M., Combined Molecular Algorithms for the Generation, Equilibration and Topological Analysis of Entangled Polymers: Methodology and Performance. *Int. J. Mol. Sci.* **2009,** *10* (11), 5054-5089.
